# Supplementary material for: Modelling Skylarks (Alauda arvensis) to Predict Impacts of Changes in Land Management and Policy: Development and Testing of an Agent-Based Model
Source: PLoS One. 2013 Jun 6;8(6):e65803. doi: 10.1371/journal.pone.0065803 (PMC3675089; doi:10.1371/journal.pone.0065803)
Supplement: Supporting Information S4 — The skylark ODdox as a zipped archive. (ZIP) [file pone.0065803.s004.zip › Skylark_ODdox/class_pesticide.html]

ALMaSS Skylark ODdox: Pesticide Class Reference


ALMaSS Skylark ODdox
 2.0


- Main Page
- Related Pages
- Classes
- Files

- Class List
- Class Index
- Class Hierarchy
- Class Members

Public Member Functions |
Protected Member Functions |
Protected Attributes

Pesticide Class Reference

`#include <pesticide.h>`

List of all members.

|  |  |
| --- | --- |
| Public Member Functions | |
| void | DailyQueueAdd (LE \*a\_element\_sprayed, double a\_amount) |
| void | DiffusionMaskInitTest (void) |
|  | Pesticide (RasterMap \*a\_land, Landscape \*a\_map) |
| bool | SavePPM (double \*a\_map, int a\_beginx, int a\_width, int a\_beginy, int a\_height, char \*a\_filename) |
| double | SupplyPesticide (int a\_x, int a\_y) |
| double | SupplyPesticide (int a\_polyref) |
| void | Test (Landscape \*a\_map) |
| void | Tick (void) |
| virtual | ~Pesticide (void) |

|  |  |
| --- | --- |
| Protected Member Functions | |
| void | DailyQueueClear (void) |
| void | DailyQueueProcess (void) |
| double | DiffusionFunction (double a\_dist\_meters) |
| void | DiffusionMaskInit (void) |
| void | DiffusionSprayPixel (int a\_x, int a\_limit\_x, int a\_y, int a\_limit\_y, double a\_amount) |
| bool | ElementIsWater (int a\_x, int a\_y) |
| void | MainMapDecay (void) |
| void | TwinMapAddToMain (void) |
| void | TwinMapClear (void) |
| void | TwinMapCopyToMask (void) |
| void | TwinMapDiffusion (void) |
| void | TwinMapSpray (LE \*a\_element\_sprayed, double a\_amount) |
| void | TwinMapSprayCorrectBorders (void) |
| void | TwinMapSprayPixel (int a\_large\_map\_x, int a\_large\_map\_y, double a\_fractional\_amount) |

|  |  |
| --- | --- |
| Protected Attributes | |
| double | m\_corr\_x |
| double | m\_corr\_y |
| vector< PesticideEvent \* > | m\_daily\_spray\_queue |
| vector< Diffusor \* > | m\_diffusion\_mask |
| RasterMap \* | m\_land |
| Landscape \* | m\_map |
| double | m\_pest\_daily\_decay\_frac |
| unsigned int | m\_pest\_map\_height |
| double \* | m\_pest\_map\_main |
| double \* | m\_pest\_map\_mask |
| unsigned int | m\_pest\_map\_size |
| double \* | m\_pest\_map\_twin |
| unsigned int | m\_pest\_map\_width |
| double | m\_prop |
| bool | m\_something\_to\_decay |
| int | m\_x\_excess |
| int | m\_y\_excess |

---

## Constructor & Destructor Documentation

|  |  |  |  |
| --- | --- | --- | --- |
| Pesticide::Pesticide | ( | RasterMap \* | *a\_land*, |
|  |  | Landscape \* | *a\_map* |
|  | ) |  |  |

References DiffusionMaskInit(), g\_msg, l\_pest\_ai\_half\_life, l\_pest\_enable\_pesticide\_engine, m\_corr\_x, m\_corr\_y, m\_land, m\_map, m\_pest\_daily\_decay\_frac, m\_pest\_map\_height, m\_pest\_map\_main, m\_pest\_map\_mask, m\_pest\_map\_size, m\_pest\_map\_twin, m\_pest\_map\_width, m\_prop, m\_something\_to\_decay, m\_x\_excess, m\_y\_excess, RasterMap::MapHeight(), RasterMap::MapWidth(), PEST\_GRIDAREA, PEST\_GRIDSIZE, PEST\_GRIDSIZE\_POW2, CfgFloat::value(), CfgBool::value(), MapErrorMsg::Warn(), and WARN\_FATAL.

{

if ( l\_pest\_enable\_pesticide\_engine.value()) {

m\_map = a\_map;

m\_land = a\_land;

m\_something\_to\_decay = false;

// Figure out critical border coordinates,

// proportional fractions etc.

m\_prop = 1.0 / ((double)(PEST\_GRIDAREA));

m\_x\_excess = m\_land->MapWidth() & (PEST\_GRIDSIZE-1);

if ( m\_x\_excess ) {

m\_corr\_x = (double)PEST\_GRIDSIZE /(double)m\_x\_excess;

}

m\_y\_excess = m\_land->MapHeight() & (PEST\_GRIDSIZE-1);

if ( m\_y\_excess ) {

m\_corr\_y = (double)PEST\_GRIDSIZE / (double)m\_y\_excess;

}

m\_pest\_map\_width = m\_land->MapWidth() >> PEST\_GRIDSIZE\_POW2;

if ( m\_land->MapWidth() & (PEST\_GRIDSIZE-1))

m\_pest\_map\_width++;

m\_pest\_map\_height = m\_land->MapHeight() >> PEST\_GRIDSIZE\_POW2;

if ( m\_land->MapHeight() & (PEST\_GRIDSIZE-1))

m\_pest\_map\_height++;

m\_pest\_map\_size = m\_pest\_map\_width \* m\_pest\_map\_height;

m\_pest\_map\_main = (double\*)malloc(sizeof(double)\* m\_pest\_map\_size );

m\_pest\_map\_twin = (double\*)malloc(sizeof(double)\* m\_pest\_map\_size );

m\_pest\_map\_mask = (double\*)malloc(sizeof(double)\* m\_pest\_map\_size );

if ( l\_pest\_enable\_pesticide\_engine.value() && ((m\_pest\_map\_twin == NULL || m\_pest\_map\_mask == NULL) || (m\_pest\_map\_main == NULL) ) ){

g\_msg->Warn(WARN\_FATAL,

"Pesticide::Pesticide(): Out of memory.", "" );

exit(1);

}

for ( unsigned int i=0; i<m\_pest\_map\_size; i++ ) {

m\_pest\_map\_main[i] = 0.0;

m\_pest\_map\_twin[i] = 0.0;

m\_pest\_map\_mask[i] = 0.0;

}

m\_pest\_daily\_decay\_frac =

pow( 10.0, log10(0.5)/l\_pest\_ai\_half\_life.value() );

DiffusionMaskInit();

}

}

|  |  |  |  |  |  |
| --- | --- | --- | --- | --- | --- |
| Pesticide::~Pesticide | ( | void |  | ) |  |

virtual

References DailyQueueClear(), l\_pest\_enable\_pesticide\_engine, m\_diffusion\_mask, m\_pest\_map\_main, m\_pest\_map\_mask, m\_pest\_map\_twin, and CfgBool::value().

{

if ( l\_pest\_enable\_pesticide\_engine.value()) {

free( m\_pest\_map\_main );

}

if ( l\_pest\_enable\_pesticide\_engine.value()) {

free( m\_pest\_map\_twin );

free( m\_pest\_map\_mask );

}

for ( unsigned int i=0; i<m\_diffusion\_mask.size(); i++ ) {

delete m\_diffusion\_mask[i];

}

m\_diffusion\_mask.resize(0);

DailyQueueClear();

}

---

## Member Function Documentation

|  |  |  |  |
| --- | --- | --- | --- |
| void Pesticide::DailyQueueAdd | ( | LE \* | *a\_element\_sprayed*, |
|  |  | double | *a\_amount* |
|  | ) |  |  |

References m\_daily\_spray\_queue.

Referenced by Orchard::DoDevelopment(), OrchardBand::DoDevelopment(), Farm::ProductApplication0(), Farm::ProductApplication1(), and Test().

{

PesticideEvent \*l\_event =

new PesticideEvent( a\_element\_sprayed, a\_amount );

m\_daily\_spray\_queue.resize( m\_daily\_spray\_queue.size() + 1 );

m\_daily\_spray\_queue[ m\_daily\_spray\_queue.size() - 1 ] =

l\_event;

}

|  |  |  |  |  |  |
| --- | --- | --- | --- | --- | --- |
| void Pesticide::DailyQueueClear | ( | void |  | ) |  |

protected

References m\_daily\_spray\_queue.

Referenced by Tick(), and ~Pesticide().

{

for ( unsigned int i=0; i<m\_daily\_spray\_queue.size(); i++ ) {

delete m\_daily\_spray\_queue[i];

}

m\_daily\_spray\_queue.resize( 0 );

}

|  |  |  |  |  |  |
| --- | --- | --- | --- | --- | --- |
| void Pesticide::DailyQueueProcess | ( | void |  | ) |  |

protected

References m\_daily\_spray\_queue, m\_something\_to\_decay, TwinMapClear(), TwinMapCopyToMask(), TwinMapDiffusion(), and TwinMapSpray().

Referenced by Tick().

{

if ( 0 == m\_daily\_spray\_queue.size() )

// Event queue empty, nobody sprayed anything today.

return;

// Spraying at least one field. Force the main pesticide map

// decay method to run tomorrow.

m\_something\_to\_decay = true;

for ( unsigned int i=0; i<m\_daily\_spray\_queue.size(); i++ ) {

TwinMapClear();

TwinMapSpray( m\_daily\_spray\_queue[i]->m\_sprayed\_elem,

m\_daily\_spray\_queue[i]->m\_amount );

TwinMapCopyToMask();

TwinMapDiffusion();

// TwinMapAddToMain(); // removal of this saves a loop through the data

}

}

|  |  |  |  |  |  |
| --- | --- | --- | --- | --- | --- |
| double Pesticide::DiffusionFunction | ( | double | *a\_dist\_meters* | ) |  |

protected

The equation provided here is the one that determines the drift of pesticides with distance. It is important that if the drift is set to zero that the result of the equation is unity.

References l\_pest\_diffusion\_slope, l\_pest\_zero\_threshold, and CfgFloat::value().

Referenced by DiffusionMaskInit().

{

double pp;

// y=(2.753767)\*(x+(1.86976))\*\*(-2.121563)

#ifdef \_\_WithinOrchardPesticideSim\_\_

if (a\_dist\_meters==0) pp=0.7784;

else pp=0.0277;

#else

// pp=2.753767 \* pow(( a\_dist\_meters+1.86976 ), -2.121563);

pp= exp( l\_pest\_diffusion\_slope.value() \* a\_dist\_meters );

if (pp<(l\_pest\_zero\_threshold.value()/10)) pp=0; // Don't bother with the little ones

#endif

return pp;

}

|  |  |  |  |  |  |
| --- | --- | --- | --- | --- | --- |
| void Pesticide::DiffusionMaskInit | ( | void |  | ) |  |

protected

References DiffusionFunction(), l\_pest\_diffusion\_grid\_count, m\_diffusion\_mask, and CfgInt::value().

Referenced by Pesticide().

{

int l\_grid = l\_pest\_diffusion\_grid\_count.value();

int l\_side\_length = l\_grid \* 2 + 1;

m\_diffusion\_mask.resize( l\_side\_length \* l\_side\_length );

unsigned int i = 0;

//double l\_diff\_zero = DiffusionFunction( 0.0 );

double l\_diff\_sum = 0.0;

for ( int x = -l\_grid; x < l\_grid + 1; x++ ) {

for ( int y = -l\_grid; y < l\_grid + 1; y++ ) {

double l\_dist = sqrt( (double) x\*x + y\*y ); //\*\*\*CJT\*\*\* added 28/8/2009

// m\_diffusion\_mask has the square to drift spray to with the proportions relative to the amount received in the centre square

// but the total may be greater than one, so we need the adjustment provided by the second loop

double l\_diff = DiffusionFunction(l\_dist);

l\_diff\_sum += l\_diff;

m\_diffusion\_mask[i] = new Diffusor( x, y, l\_diff); // \*\*\*CJT\*\*\* changed 28/8/2009 l\_diff\_zero

i++;

}

}

for ( unsigned x = 0; x < i; x++) {

m\_diffusion\_mask[x]->m\_fraction /= l\_diff\_sum;

}

}

|  |  |  |  |  |  |
| --- | --- | --- | --- | --- | --- |
| void Pesticide::DiffusionMaskInitTest | ( | void |  | ) |  |

References l\_pest\_diffusion\_grid\_count, m\_diffusion\_mask, and CfgInt::value().

{

int l\_grid = l\_pest\_diffusion\_grid\_count.value();

unsigned int i = 0;

for ( int x = -l\_grid; x < l\_grid + 1; x++ ) {

for ( int y = -l\_grid; y < l\_grid + 1; y++ ) {

printf( "%2.4f ", m\_diffusion\_mask[i]->m\_fraction );

i++;

}

printf("\n");

}

}

|  |  |  |  |
| --- | --- | --- | --- |
| void Pesticide::DiffusionSprayPixel | ( | int | *a\_x*, |
|  |  | int | *a\_limit\_x*, |
|  |  | int | *a\_y*, |
|  |  | int | *a\_limit\_y*, |
|  |  | double | *a\_amount* |
|  | ) |  |  |

inlineprotected

References m\_pest\_map\_main.

Referenced by TwinMapDiffusion().

{

if ( a\_x < 0 ||

a\_x >= a\_limit\_x ||

a\_y < 0 ||

a\_y >= a\_limit\_y

)

return;

int l\_coord = a\_y \* a\_limit\_x + a\_x;

/\* \*\*\*CJT\*\*\* changed 28/8/2009

if ( m\_pest\_map\_twin[ l\_coord ] < a\_amount ) {

m\_pest\_map\_twin[ l\_coord ] = a\_amount;

}

\*/

m\_pest\_map\_main[ l\_coord ] += a\_amount; //\*\*\*CJT\*\*\* changed 28/8/2009

}

|  |  |  |  |
| --- | --- | --- | --- |
| bool Pesticide::ElementIsWater | ( | int | *a\_x*, |
|  |  | int | *a\_y* |
|  | ) |  |  |

inlineprotected

References m\_map, Landscape::SupplyElementType(), tole\_Freshwater, tole\_River, and tole\_Saltwater.

Referenced by SupplyPesticide().

{

TTypesOfLandscapeElement l\_eletype =

m\_map->SupplyElementType( a\_x, a\_y );

if ( l\_eletype == tole\_Freshwater ||

l\_eletype == tole\_River ||

l\_eletype == tole\_Saltwater

)

return true;

return false;

}

|  |  |  |  |  |  |
| --- | --- | --- | --- | --- | --- |
| void Pesticide::MainMapDecay | ( | void |  | ) |  |

protected

References l\_pest\_zero\_threshold, m\_pest\_daily\_decay\_frac, m\_pest\_map\_main, m\_pest\_map\_size, m\_something\_to\_decay, and CfgFloat::value().

Referenced by Tick().

{

if ( ! m\_something\_to\_decay )

return;

double l\_zero = l\_pest\_zero\_threshold.value();

m\_something\_to\_decay = false;

for ( unsigned int i=0; i<m\_pest\_map\_size; i++ ) {

if ( m\_pest\_map\_main[i] > l\_zero ) {

m\_pest\_map\_main[i] \*= m\_pest\_daily\_decay\_frac;

m\_something\_to\_decay = true;

} else {

m\_pest\_map\_main[i] = 0.0;

}

}

}

|  |  |  |  |
| --- | --- | --- | --- |
| bool Pesticide::SavePPM | ( | double \* | *a\_map*, |
|  |  | int | *a\_beginx*, |
|  |  | int | *a\_width*, |
|  |  | int | *a\_beginy*, |
|  |  | int | *a\_height*, |
|  |  | char \* | *a\_filename* |
|  | ) |  |  |

References g\_msg, m\_pest\_map\_height, m\_pest\_map\_width, SV\_UINT32, SV\_UINT8, MapErrorMsg::Warn(), and WARN\_FATAL.

Referenced by Test().

{

a\_beginx = 0;

a\_width = m\_pest\_map\_width;

a\_beginy = 0;

a\_height = m\_pest\_map\_height;

SV\_UINT32 linesize = a\_width\*3;

SV\_UINT8\* linebuffer = (SV\_UINT8\*)malloc(sizeof(SV\_UINT8)\* linesize);

if ( linebuffer == NULL ) {

g\_msg->Warn( WARN\_FATAL,

"Pesticide::SavePPM(): Out of memory!", "" );

exit(1);

}

FILE\* l\_file;

l\_file=fopen(a\_filename, "w" );

if ( !l\_file ) {

printf("PesticideTest::SavePPM(): "

"Unable to open file for writing: %s\n",

a\_filename );

exit(1);

}

fprintf( l\_file, "P6\n%d %d %d\n",

a\_width,

a\_height,

255 );

for ( int line=a\_beginy; line< a\_beginy + a\_height; line++ ) {

int i = 0;

for ( int column=a\_beginx; column < a\_beginx + a\_width; column++ ) {

int localcolor = (int)( a\_map[ line \* m\_pest\_map\_width + column ]

\* 255.0);

if ( localcolor <= 255 ) {

linebuffer [ i++ ] = char (localcolor & 0xff);

linebuffer [ i++ ] = 0;

linebuffer [ i++ ] = 0;

} else {

linebuffer [ i++ ] = 255;

localcolor -= 255;

if ( localcolor <= 255 ) {

linebuffer [ i++ ] = char (localcolor);

linebuffer [ i++ ] = 0;

} else {

linebuffer [ i++ ] = 255;

localcolor -= 255;

if ( localcolor <= 255 ) {

linebuffer [ i++ ] = char (localcolor);

} else {

linebuffer [ i++ ] = 255;

}

}

}

}

fwrite( linebuffer, sizeof(SV\_UINT8), linesize, l\_file );

}

fclose( l\_file );

free( linebuffer );

return true;

}

|  |  |  |  |
| --- | --- | --- | --- |
| double Pesticide::SupplyPesticide | ( | int | *a\_x*, |
|  |  | int | *a\_y* |
|  | ) |  |  |

inline

Parameters:
:   |  |  |
    | --- | --- |
    | a\_x | the x-coord in landscape x units |
    | a\_y | the y-coord in landscape x units |

Returns:
:   Returns the value of the pesticide grid cell containing a\_x,a\_y

References ElementIsWater(), m\_pest\_map\_main, m\_pest\_map\_width, and PEST\_GRIDSIZE\_POW2.

Referenced by Landscape::SupplyPesticide().

{

#ifdef PEST\_WATER\_CHECK

if ( ElementIsWater( a\_x, a\_y ))

return 0.0;

#endif

int l\_x = a\_x >> PEST\_GRIDSIZE\_POW2;

int l\_y = a\_y >> PEST\_GRIDSIZE\_POW2;

return m\_pest\_map\_main[ l\_y \* m\_pest\_map\_width + l\_x ];

}

|  |  |  |  |  |  |
| --- | --- | --- | --- | --- | --- |
| double Pesticide::SupplyPesticide | ( | int | *a\_polyref* | ) |  |

inline

References m\_map, m\_pest\_map\_main, and Landscape::SupplyPesticideCell().

{

// This is an approximation because we have no idea about the actual variation

// in pesticide concentrations within the polygon.

int l\_c = m\_map->SupplyPesticideCell(a\_ele);

return m\_pest\_map\_main[ l\_c ];

}

|  |  |  |  |  |  |
| --- | --- | --- | --- | --- | --- |
| void Pesticide::Test | ( | Landscape \* | *a\_map* | ) |  |

References DailyQueueAdd(), m\_pest\_map\_main, random(), SavePPM(), Landscape::SupplyPolyRef(), and Tick().

{

for ( int i=0; i<730; i++) {

if ( random(100) < 10 ) {

int l\_sprays = random(5)+1;

for ( int j=0; j<l\_sprays; j++ ) {

int l\_x = random(1500);

int l\_y = random(1500);

DailyQueueAdd( a\_map->

SupplyLEPointer( a\_map->SupplyPolyRef( l\_x, l\_y )),

1.0 );

}

char l\_filename[20];

sprintf( l\_filename, "ppms/p%04d.ppm", i );

SavePPM( m\_pest\_map\_main,

0, 0, 0, 0,

l\_filename );

}

Tick();

}

}

|  |  |  |  |  |  |
| --- | --- | --- | --- | --- | --- |
| void Pesticide::Tick | ( | void |  | ) |  |

References DailyQueueClear(), DailyQueueProcess(), l\_pest\_enable\_pesticide\_engine, MainMapDecay(), and CfgBool::value().

Referenced by Test(), and Landscape::Tick().

{

if ( ! l\_pest\_enable\_pesticide\_engine.value()) {

DailyQueueClear();

return;

}

MainMapDecay();

DailyQueueProcess();

DailyQueueClear();

}

|  |  |  |  |  |  |
| --- | --- | --- | --- | --- | --- |
| void Pesticide::TwinMapAddToMain | ( | void |  | ) |  |

protected

References m\_pest\_map\_main, m\_pest\_map\_size, and m\_pest\_map\_twin.

{

for ( unsigned int i=0; i<m\_pest\_map\_size; i++ ) {

m\_pest\_map\_main[i] += m\_pest\_map\_twin[i];

}

}

|  |  |  |  |  |  |
| --- | --- | --- | --- | --- | --- |
| void Pesticide::TwinMapClear | ( | void |  | ) |  |

protected

References m\_pest\_map\_size, and m\_pest\_map\_twin.

Referenced by DailyQueueProcess().

{

for ( unsigned int i=0; i<m\_pest\_map\_size; i++ ) {

m\_pest\_map\_twin[i] = 0.0;

}

}

|  |  |  |  |  |  |
| --- | --- | --- | --- | --- | --- |
| void Pesticide::TwinMapCopyToMask | ( | void |  | ) |  |

protected

References m\_pest\_map\_mask, m\_pest\_map\_size, and m\_pest\_map\_twin.

Referenced by DailyQueueProcess().

{

// Copy the newly calculated pesticide amounts over to the main map

for ( unsigned int i=0; i<m\_pest\_map\_size; i++ ) {

m\_pest\_map\_mask[i] = m\_pest\_map\_twin[i];

}

}

|  |  |  |  |  |  |
| --- | --- | --- | --- | --- | --- |
| void Pesticide::TwinMapDiffusion | ( | void |  | ) |  |

protected

References DiffusionSprayPixel(), m\_diffusion\_mask, m\_pest\_map\_height, m\_pest\_map\_mask, and m\_pest\_map\_width.

Referenced by DailyQueueProcess().

{

int l\_width = (int)m\_pest\_map\_width;

int l\_height = (int)m\_pest\_map\_height;

for ( int x=0; x<l\_width; x++ ) {

for ( int y=0; y<l\_height; y++ ) {

if ( m\_pest\_map\_mask[ y\*l\_width + x ] > 0.0 ) {

double l\_amount = m\_pest\_map\_mask[ y\*l\_width + x ];

for ( unsigned int i=0; i<m\_diffusion\_mask.size(); i++ ) {

DiffusionSprayPixel( x + m\_diffusion\_mask[i]->m\_dx,l\_width,y + m\_diffusion\_mask[i]->m\_dy,l\_height,m\_diffusion\_mask[i]->m\_fraction \*l\_amount);

}

}

}

}

}

|  |  |  |  |
| --- | --- | --- | --- |
| void Pesticide::TwinMapSpray | ( | LE \* | *a\_element\_sprayed*, |
|  |  | double | *a\_amount* |
|  | ) |  |  |

protected

References RasterMap::Get(), LE::GetMapIndex(), LE::GetMaxX(), LE::GetMaxY(), LE::GetMinX(), LE::GetMinY(), LE::GetVegBiomass(), LE::GetVegCover(), l\_pest\_use\_application\_rate, m\_land, m\_prop, TwinMapSprayCorrectBorders(), TwinMapSprayPixel(), and CfgBool::value().

Referenced by DailyQueueProcess().

{

if (l\_pest\_use\_application\_rate.value()) {

// We are applying a field rate. The actual residue needs to be calculated here

double biomass = a\_element\_sprayed->GetVegBiomass();

double cover = a\_element\_sprayed->GetVegCover();

// rate is in mg/m and needs to be converted to mg/kg

double residue = (a\_amount/(biomass\*0.001)) \* 0.435855; // g to kg veg // 0.435855 is a specific calculation to obtain the same residue that Joe Crocker used for the scale of use study

a\_amount = residue \* cover;

}

double l\_fractional\_amount = a\_amount \* m\_prop;

int l\_large\_map\_index = a\_element\_sprayed->GetMapIndex();

// Going through the whole landscape is very slow and unnecessary for small polygons

// Since our polygons do not extend beyond the edge of the map

// ie do not wrap round, then we only need a measure of minx, maxx, miny, maxy

// this is set up at the start of the simulation.

int minx=a\_element\_sprayed->GetMinX();

int maxx=a\_element\_sprayed->GetMaxX();

int miny=a\_element\_sprayed->GetMinY();

int maxy=a\_element\_sprayed->GetMaxY();

for ( int y=miny; y<=maxy; y++ ) {

for ( int x=minx; x<=maxx; x++ ) {

if ( m\_land->Get( x, y ) == l\_large\_map\_index ) {

TwinMapSprayPixel( x, y, l\_fractional\_amount );

}

}

}

TwinMapSprayCorrectBorders();

}

|  |  |  |  |  |  |
| --- | --- | --- | --- | --- | --- |
| void Pesticide::TwinMapSprayCorrectBorders | ( | void |  | ) |  |

protected

References m\_corr\_x, m\_corr\_y, m\_pest\_map\_height, m\_pest\_map\_size, m\_pest\_map\_twin, m\_pest\_map\_width, m\_x\_excess, m\_y\_excess, and PEST\_GRIDAREA.

Referenced by TwinMapSpray().

{

if ( m\_x\_excess ) {

for ( unsigned int i=0; i<m\_pest\_map\_width-1; i++ ) {

m\_pest\_map\_twin[ i \* m\_pest\_map\_width + m\_pest\_map\_height-1 ] \*=

m\_corr\_x;

}

}

if ( m\_y\_excess ) {

unsigned int l\_additive = (m\_pest\_map\_height-1)\*m\_pest\_map\_width;

for ( unsigned int i=0; i<m\_pest\_map\_height-1; i++ ) {

m\_pest\_map\_twin[ i + l\_additive ] \*=

m\_corr\_y;

}

}

if ( m\_x\_excess && m\_y\_excess ) {

m\_pest\_map\_twin[ m\_pest\_map\_size-1 ] \*= (((double)(PEST\_GRIDAREA)) / ((double)(m\_x\_excess\*m\_y\_excess)));

}

}

|  |  |  |  |
| --- | --- | --- | --- |
| void Pesticide::TwinMapSprayPixel | ( | int | *a\_large\_map\_x*, |
|  |  | int | *a\_large\_map\_y*, |
|  |  | double | *a\_fractional\_amount* |
|  | ) |  |  |

inlineprotected

References m\_pest\_map\_twin, m\_pest\_map\_width, and PEST\_GRIDSIZE\_POW2.

Referenced by TwinMapSpray().

{

int l\_x = a\_large\_map\_x >> PEST\_GRIDSIZE\_POW2;

int l\_y = a\_large\_map\_y >> PEST\_GRIDSIZE\_POW2;

m\_pest\_map\_twin[ l\_y \* m\_pest\_map\_width + l\_x ] +=

a\_fractional\_amount;

}

---

## Member Data Documentation

double Pesticide::m\_corr\_x

protected

Referenced by Pesticide(), and TwinMapSprayCorrectBorders().

double Pesticide::m\_corr\_y

protected

Referenced by Pesticide(), and TwinMapSprayCorrectBorders().

vector<PesticideEvent\*> Pesticide::m\_daily\_spray\_queue

protected

Referenced by DailyQueueAdd(), DailyQueueClear(), and DailyQueueProcess().

vector<Diffusor\*> Pesticide::m\_diffusion\_mask

protected

Referenced by DiffusionMaskInit(), DiffusionMaskInitTest(), TwinMapDiffusion(), and ~Pesticide().

RasterMap\* Pesticide::m\_land

protected

Referenced by Pesticide(), and TwinMapSpray().

Landscape\* Pesticide::m\_map

protected

Referenced by ElementIsWater(), Pesticide(), and SupplyPesticide().

double Pesticide::m\_pest\_daily\_decay\_frac

protected

Referenced by MainMapDecay(), and Pesticide().

unsigned int Pesticide::m\_pest\_map\_height

protected

Referenced by Pesticide(), SavePPM(), TwinMapDiffusion(), and TwinMapSprayCorrectBorders().

double\* Pesticide::m\_pest\_map\_main

protected

Referenced by DiffusionSprayPixel(), MainMapDecay(), Pesticide(), SupplyPesticide(), Test(), TwinMapAddToMain(), and ~Pesticide().

double\* Pesticide::m\_pest\_map\_mask

protected

Referenced by Pesticide(), TwinMapCopyToMask(), TwinMapDiffusion(), and ~Pesticide().

unsigned int Pesticide::m\_pest\_map\_size

protected

Referenced by MainMapDecay(), Pesticide(), TwinMapAddToMain(), TwinMapClear(), TwinMapCopyToMask(), and TwinMapSprayCorrectBorders().

double\* Pesticide::m\_pest\_map\_twin

protected

Referenced by Pesticide(), TwinMapAddToMain(), TwinMapClear(), TwinMapCopyToMask(), TwinMapSprayCorrectBorders(), TwinMapSprayPixel(), and ~Pesticide().

unsigned int Pesticide::m\_pest\_map\_width

protected

Referenced by Pesticide(), SavePPM(), SupplyPesticide(), TwinMapDiffusion(), TwinMapSprayCorrectBorders(), and TwinMapSprayPixel().

double Pesticide::m\_prop

protected

Referenced by Pesticide(), and TwinMapSpray().

bool Pesticide::m\_something\_to\_decay

protected

Referenced by DailyQueueProcess(), MainMapDecay(), and Pesticide().

int Pesticide::m\_x\_excess

protected

Referenced by Pesticide(), and TwinMapSprayCorrectBorders().

int Pesticide::m\_y\_excess

protected

Referenced by Pesticide(), and TwinMapSprayCorrectBorders().

---

The documentation for this class was generated from the following files:

- pesticide.h
- pesticide.cpp


- Pesticide
- Generated on Thu Jan 10 2013 13:15:36 for ALMaSS Skylark ODdox by
   1.8.1.1
